# 10 kHz spinal cord stimulation vs. traditional low-frequency spinal cord stimulation for the treatment of diabetes peripheral neuropathic pain: study protocol for a multi-center randomized controlled clinical trial

**DOI:** 10.3389/fneur.2025.1611970

**Published:** 2025-06-04

**Authors:** Chen Li, Chun-Hua Liu, Yi-Fan Li, Hui-Min Hu, Qing Shi, An-Xiang Liu, Wen-Hui Liu, Yi Zhang, Peng Mao, Bi-Fa Fan

**Affiliations:** ^1^Graduate School, Beijing University of Chinese Medicine, Beijing, China; ^2^Department of Pain Medicine, China-Japan Friendship Hospital, Beijing, China

**Keywords:** diabetic peripheral neuropathic pain, high-frequency spinal cord stimulation, randomized controlled trial, neuromodulation, protocol

## Abstract

**Background:**

Diabetic peripheral neuropathic pain (DPNP), affecting ~50% of diabetes patients, imposes major burdens on quality of life and healthcare systems, while current therapies including pharmacotherapy and conventional spinal cord stimulation remain limited by insufficient efficacy and adverse effects. Our study aims to evaluate the clinical efficacy and safety of 10 kHz high-frequency spinal cord stimulation (HF-SCS) compared to traditional low-frequency SCS (T-SCS) in alleviating DPNP.

**Methods:**

This prospective, randomized, controlled, multicenter trial will enroll 100 participants with DPNP. Patients aged 18–80 with chronic (≥6-month) lower limb pain will be randomly assigned to HF-SCS (10 kHz) vs. T-SCS (40–60 Hz). The primary outcome is the treatment efficacy rate, defined as ≥50% reduction in numeric rating scale (NRS) scores at 3 months post-intervention. Secondary outcomes include improvements in quality of life (Short Form 12), sleep quality (Athens Insomnia Scale), psychological status (Beck Depression Inventory), neuropathy severity (Michigan Neuropathy Screening Instrument), and microcirculatory parameters assessed via infrared thermography. Safety evaluations encompass adverse events, laboratory tests, and imaging findings.

**Discussion:**

This study seeks to provide robust evidence on the superiority of HF-SCS in pain relief, functional improvement, and microcirculatory benefits, potentially establishing it as a preferred neuromodulation strategy for DPNP. Findings may advance clinical practice by addressing unmet needs in chronic pain management through targeted, mechanism-driven interventions.

**Clinical trial registration:**

https://www.chictr.org.cn/indexEN.html, identifier: ChiCTR2300078291.

## 1 Introduction

Global diabetes prevalence is projected to increase from 9.3% in 2019 to 10.2% by 2030 and further to 10.9% by 2045 (1). Diabetic peripheral neuropathic pain (DPNP) is a common complication affecting ~50% of individuals with diabetes (2). This condition not only severely impacts patients' quality of life, causing sleep disturbances, depression, and anxiety, but also increases the overall healthcare burden due to high treatment costs (3).

Currently, there is no definitive and effective treatment for DPNP. Conventional therapeutic modalities—including pharmacotherapy, lumbar sympathetic nerve block, and alternative therapies—are typically associated with limited efficacy and frequent side effects (4–6). Furthermore, the complex pathophysiological changes induced by primary diabetes, including oxidative stress, vascular ischemia and hypoxia, and neurotrophic factor deficiency, pose significant challenges for both basic and clinical research on DPNP (7, 8). As a result, targeted treatments for DPNP have yet to achieve significant breakthroughs, making it one of the most pressing clinical challenges.

Spinal cord stimulation (SCS) is an internationally recognized and widely used method for treating chronic refractory pain (9). Traditional SCS (T-SCS) therapy involves implanting stimulating electrodes in the epidural space and delivering low-frequency electrical stimulation at 40–60 Hz to the spinal cord segments corresponding to the pain region. This stimulation produces a tingling sensation, which effectively masks the pain and provides analgesic relief. Numerous clinical studies have demonstrated the efficacy of T-SCS in treating DPNP, confirming its potential as a treatment option (10–12). T-SCS operates based on the Gate Control Theory, which activates amyloid β (Aβ) fibers in the dorsal horn through electrical pulses (13). This activation promotes inhibitory interneuron activity and increases the release of the inhibitory neurotransmitter γ-aminobutyric acid (GABA), which helps block nociceptive signal transmission (14). However, over time, T-SCS often leads to paresthesia and a diminished analgesic effect, limiting its long-term effectiveness (15).

Recent advances in SCS have facilitated the development of high-frequency spinal cord stimulation (HF-SCS). Basic experimental results demonstrate that HF-SCS can reduce mechanical sensitivity with long-lasting analgesic effects after cessation in peripheral nerve injury animal models (16). Additionally, HF-SCS significantly increases vasodilation compared to normal-frequency SCS, potentially through the retrograde stimulation of unmyelinated C fibers and the induced release of calcitonin gene-related peptides (17). HF-SCS mechanisms directly target DPNP pathogenesis, establishing a pathophysiological rationale for clinical use. Based on this, our study aims to evaluate HF-SCS vs. T-SCS for DPNP, providing evidence to support HF-SCS clinical application.

## 2 Methods and analysis

### 2.1 Study design

**Figure 1:**
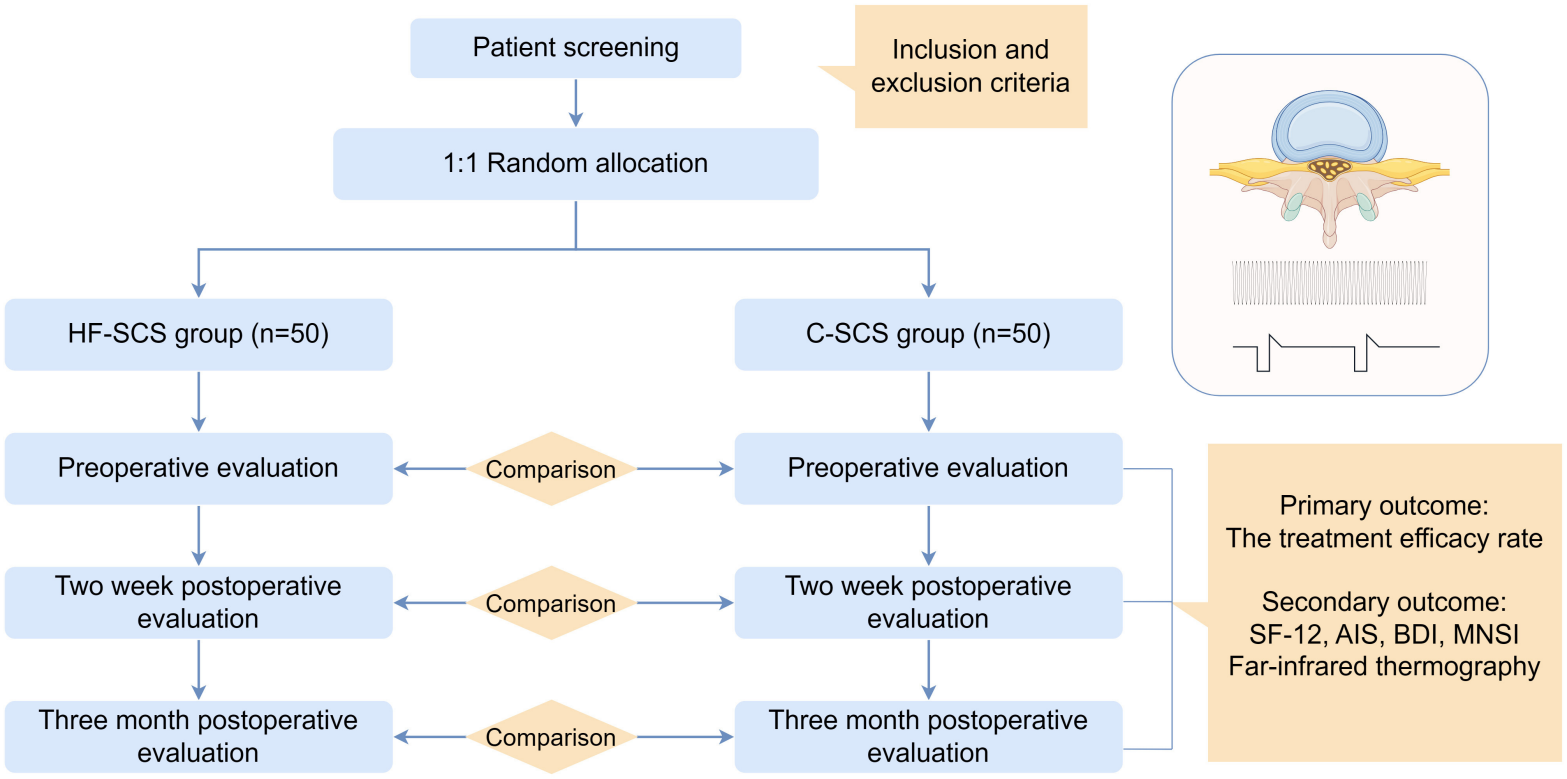
Flowchart of the study.

This prospective randomized controlled multicenter study will enroll 100 participants recruited competitively from multiple centers, with equal randomization (*n* = 50/group) to either HF-SCS (H-group) or SCS (C-group) therapy. The trial comprises three phases: baseline evaluation, 10–14-day intervention period, and 3-month post-operation follow-up. The flow chart is shown in Figure 1 by Figdraw. All participant characteristics and clinical outcomes will be documented using case report forms (CRFs). Data collection procedures are outlined in Table 1.

**Table 1:** Schedule of data collection.

**Procedure**	**Screening period**	**Intervention**	**Follow-up period**	
	**Visit1**		**Visit2**	**Vist3**
	**Day−7 to 0**	**Day 0**	**Day 14 ±5**	**Day 90 ±7**
Informed consent	X			
Demographic information	X			
Physical examination	X		X	X
Vital signs	X		X	X
Vertebral X-ray	X		X	
ECG	X		X	
NRS	X		X	X
MNSI	X		X	X
SF-12	X		X	X
AIS	X		X	X
BDI	X		X	X
Glycosylated hemoglobin	X			
Insulin and C-peptide	X			
Routine blood test	X		X	
Liver and kidney function	X		X	
Lower limb infrared thermography	X		X	X
Randomization	X			
Surgical operation		X		
Adverse event	X		X	X
Combined medication	X		X	X
Treatment records	X		X	X

ECG, Electrocardiogram; NRS, Numerical Rating Scale; MNSI, Michigan Neuropathy Screening Instrument; SF-12, 12-Item Short Form Health Survey; AIS, Athens Insomnia Scale; Beck, Beck Depression Inventory.

### 2.2 Sample size

According to the results of RCT studies comparing HF-SCS with traditional SCS for the treatment of PDPN, the efficacy rate of HF-SCS for PDPN is ~86%. Based on the statistical sample size calculation method for superiority trials, with a one-sided significance level (α) of 0.025, a test power (1-β) of 0.8, and an equal sample size ratio between the treatment group and the control group, the required sample size for each group was calculated using PASS 17.0 to be 45 cases. Considering a 10% loss to follow-up and refusal rate, at least 50 cases are needed in the treatment group and 50 cases in the control group, resulting in a total sample size of 100 cases.

### 2.3 Inclusion criteria

Participants who meet all the following requirements will be enrolled:

(1) Diagnosed with diabetes mellitus, aged between 18 and 80 years.
(2) Symmetrical distal pain in the lower limbs, with or without dysesthesia.
(3) PDPN duration >6 months.
(4) Pain characterized as prickling, electric shock-like, and/or burning sensations.
(5) Abnormal results in Quantitative Sensory Testing (QST).
(6) Presence of hyperalgesia and/or allodynia.
(7) No abnormality in lower limb reflexes and muscle strength.
(8) No spinal canal abnormalities (e.g., stenosis) on MRI or CT imaging.

### 2.4 Exclusion criteria

Participants who meet one of the following requirements will be excluded:

(1) Concomitant severe cardiovascular or cerebrovascular diseases.
(2) History of lumbar spine surgery or traumatic spinal canal stenosis within the past 6 months, or prior lumbar surgery/traumatic spinal canal stenosis that may interfere with spinal cord stimulation (SCS) procedures or pain assessment in this study.
(3) Presence of radicular symptoms.
(4) Other spinal abnormalities (e.g., benign/malignant tumors, congenital spinal anomalies, spondylolisthesis).
(5) Coagulation disorders, malignancy, active infection, or psychiatric disorders.
(6) Pregnancy.

### 2.5 Criteria for discontinuation

During this trial, subjects experiencing the following conditions will be considered as dropout cases, and the reason and date of dropout will be recorded in a case report form.

(1) Violation of inclusion/exclusion criteria at enrollment, with the investigator determining compromised participant safety.
(2) Participant intolerance to the treatment regimen.
(3) Adverse event (AE) occurrence leading to investigator-determined unsuitability for continued study treatment.
(4) Major protocol deviations (e.g., non-compliance with study procedures) affecting safety or efficacy assessments.
(5) Pregnancy.
(6) Withdrawal of informed consent: if a participant withdraws consent; the investigator must document the scope of withdrawal (e.g., discontinuation of treatment only vs. withdrawal from all study procedures/follow-ups) in medical records.
(7) Loss to follow-up: defined as failure to attend visits and inability to contact the participant despite ≥2 documented contact attempts at ≥1-week intervals.
(8) Early termination of study treatment: discontinuation of treatment while continuing study procedures/follow-ups (if applicable).
(9) Early study withdrawal: complete discontinuation of all study-related activities, including treatment and follow-ups (e.g., due to consent withdrawal or loss to follow-up).
(10) Follow-up for AE/SAE-related withdrawals: participants withdrawing due to treatment-emergent adverse events (TEAEs) or serious adverse events (SAEs) must be followed until resolution (return to baseline/stable status) or until the AE is deemed clinically insignificant by the investigator.

### 2.6 Randomization and blinding

Competitive enrollment will be conducted across centers. An independent statistician at each center will perform centralized randomization using a validated centralized randomization system. Participants will be randomly assigned to either the HF-SCS group or the T-SCS group at a 1:1 allocation ratio, based on a computer-generated random number.

Due to the distinct sensory profiles between HF-SCS and T-SCS, effective blinding of participants and outcome assessors was not feasible; therefore, only data statisticians were maintained under blinded conditions throughout the study.

## 3 Procedure

### 3.1 Surgical procedure

Participants will undergo the following standardized surgical protocol in the operating room: multiparameter physiological monitoring will be initiated, and intravenous access will be established. The participant will be positioned prone, followed by standard antiseptic preparation and draping. Under C-arm fluoroscopic guidance, the target intervertebral space corresponding to the spinal nerve innervating the painful area will be identified. After achieving satisfactory local anesthesia, an epidural puncture will be performed via a lateral approach. The needle will be advanced through the interlaminar space into the epidural space. A spinal cord stimulation (SCS) electrode will be implanted, followed by intraoperative external SCS testing to confirm paresthesia coverage overlapping the painful area. The electrode lead will be secured at the puncture site with sutures and covered with sterile dressing. The electrode extension cable will be connected to an external pulse generator.

### 3.2 Postoperative management

Participants will return to the ward for stimulation parameter adjustment (HF-SCS group: 10 kHz; T-SCS group: 40–60 Hz). Postoperative instructions will include avoidance of movements that may lead to electrode displacement (e.g., heavy lifting, overhead arm motions, hyperextension). The electrode will remain in place for 10–14 days under the therapeutic stimulation mode before removal, marking the end of the treatment phase.

## 4 Outcome

### 4.1 Primary outcome

The treatment efficacy rate, defined as a reduction of ≥50% in the numeric rating scale (NRS) score at 3 months post-treatment compared to the pre-treatment score, will be evaluated as primary outcomes.

### 4.2 Secondary outcome

#### 4.2.1 Clinical symptom assessment indicators

Short Form 12 (SF-12): evaluates the patient's quality of life.

Athens Insomnia Scale (AIS): assesses the patient's sleep condition.

Beck Depression Inventory (BDI): evaluates the patient's psychological improvement.

Michigan Neuropathy Screening Instrument (MNSI): assesses the improvement of diabetic peripheral neuropathy.

#### 4.2.2 Clinical examination assessment indicators

Before enrollment, assessments of glycated hemoglobin (HbA1c), insulin levels, and C-peptide levels are conducted to evaluate changes in the primary disease, diabetes. Additionally, bilateral lower limb infrared thermography will be performed preoperatively, at 2 weeks postoperatively, and at 3-month follow-up to assess microcirculatory function. All scans will be conducted in a dedicated temperature-controlled examination room, with the device maintained at ~1 meter from the subject and the temperature range set at 25°C−40°C to ensure optimal thermal imaging quality. Patients are also instructed to record their concurrent use of analgesic medications.

## 5 Safety evaluation

Routine blood tests, liver and kidney function tests, electrocardiograms (ECG), and X-ray imaging are performed at screening period (visit 1) and at the 2-week follow-up post-operation (visit 2). At the 3-month follow-up post-operation (visit 3), the patient's vital signs (blood pressure, heart rate, body temperature, and respiration) and physical examination are assessed. If abnormalities are detected during follow-up, the patient's clinical manifestations should be evaluated by a relevant specialist. If confirmed to be caused by the study, it should be documented as an adverse event, and appropriate treatment should be provided. Additionally, any complications related to the study (e.g., infection, cerebrospinal fluid leak, pneumothorax, electrode fracture) should be documented as adverse events, and appropriate treatment should be administered. During the trial, any serious adverse events will be promptly reported to both the medical device clinical trial administration department and the ethics committee. The study will be suspended if the trial places participants at immediate risk of life-threatening conditions or if the incidence of serious adverse events significantly exceeds expectations.

## 6 Statistical analysis

Primary evaluation indicator, the treatment efficacy rate is expressed as the percentage of patients with effective treatment. Between-group comparisons are conducted using the χ^2^ test. Secondary Evaluation Indicators, including the scores of the various scales mentioned above and the values of clinical test items, expressed as mean ± standard deviation. For normally distributed data with equal variance, an independent sample *t*-test is used. For data that are not normally distributed or have unequal variance, the rank-sum test is applied. All data are analyzed using SPSS 26.0 statistical software, with a significance level of *P* < 0.05 considered statistically significant.

## 7 Discussion

To our knowledge, this study represents one of the first attempts in China to evaluate the clinical efficacy and safety of HF-SCS in comparison to T-SCS for DPNP treatment in a randomized, controlled, multicenter clinical trial setting. This research is expected to provide significant insights into the optimization of SCS techniques for effective management of DPNP, potentially enhancing patient outcomes and informing clinical decision-making.

DPNP is a chronic complication of diabetes mellitus characterized by persistent neuropathic pain, primarily affecting the peripheral nervous system in the lower limbs (18). The pain typically manifests as burning sensations, electric shock-like sensations, tingling, and stabbing pain, severely impairing patients' quality of life and increasing healthcare expenditures (19). The pathophysiology of DPNP is complex and involves multiple factors. Persistent high blood glucose levels lead to metabolic and microvascular alterations that cause nerve ischemia, oxidative stress, increased nerve excitability, central sensitization, and diminished inhibitory modulation, ultimately resulting in chronic pain and sensory dysfunction (20). Due to its pathophysiology involving vascular, metabolic, and neuroinflammatory mechanisms, effective, and safe targeted treatments remain an urgent clinical need.

Animal studies indicated that HF-SCS significantly reduces neuroinflammation and provides long-lasting pain relief (21). Previous clinical trials also demonstrated that HF-SCS not only offers significant pain relief but also proves to be cost-effective in diverse neuropathic pain conditions, including failed back surgery syndrome and postherpetic neuralgia (22–24). Moreover, patient preference for HF-SCS has been reported, predominantly due to paresthesia-free analgesia, reduced discomfort, and higher acceptability (25, 26). If our study shows similar efficacy, it will provide further justification for the broader implementation of HF-SCS, enhancing patient adherence and satisfaction with this therapy.

In this study, we adopted rigorous methodological strategies to enhance the reliability and validity of our findings. First, randomization and blinding procedures were employed to minimize potential bias and confounding factors. Second, comprehensive outcome measures, including primary efficacy parameters (such as significant reduction in Numeric Rating Scale pain scores) and multidimensional secondary endpoints—such as quality of life (SF-12), psychological status (Beck Depression Inventory), sleep quality (Athens Insomnia Scale), and neurological function improvement (Michigan Neuropathy Screening Instrument)—were selected to capture holistic therapeutic effects on patients with DPNP. Furthermore, the protocol innovatively integrates infrared thermography as an objective assessment modality to holistically evaluate physiological alterations following clinical symptom resolution. Infrared thermography can assess abnormal thermal distribution and temperature differences in various medical conditions caused by alterations in peripheral cutaneous circulation (27). DPNP is closely associated with microcirculatory alterations. HF-SCS's proposed ability to improve microcirculation and nerve health potentially differentiates it from traditional, symptom-oriented interventions. If our findings confirm significant improvements in clinical symptoms, quality of life, and microcirculatory parameters with HF-SCS, it would strongly support its adoption as a superior treatment modality for DPNP.

In conclusion, this randomized controlled trial is anticipated to provide robust clinical evidence regarding the comparative efficacy, safety, and potential pathophysiological benefits of 10 kHz HF-SCS relative to traditional low-frequency SCS for patients suffering from refractory DPNP. Positive outcomes from this trial would signify an important advancement in chronic pain management, potentially shifting clinical practice toward a more effective and patient-preferred neuromodulation strategy.

## 8 Limitations

This study also has limitations. First, although the study was rigorously designed, potential biases from patient expectations and placebo effects remain, particularly due to differing paresthesia perceptions between HF-SCS and T-SCS. Therefore, infrared thermography will serve as an objective biomarker to complement clinical assessments. Second, due to resource constraints, our follow-up period was set at 3 months. While this duration is sufficient to assess short-term therapeutic effects, it may not fully evaluate long-term treatment outcomes. Third, the stringent inclusion criteria may restrict the generalizability of our findings to broader DPNP populations. Therefore, real-world studies with extended follow-up and expanded patient populations will be conducted in the future.

## References

[1] Saeedi P Petersohn I Salpea P Malanda B Karuranga S Unwin N . Global and regional diabetes prevalence estimates for 2019 and projections for 2030 and 2045: results from the international diabetes federation diabetes atlas, 9(th) edition. Diabetes Res Clin Pract. (2019) 157:107843. 10.1016/j.diabres.2019.107843 31518657

[2] Pop-Busui R Patel A Sang CN Banks PL Pierce PF Sun F . Efficacy and safety of LX9211 for relief of diabetic peripheral neuropathic pain (RELIEF-DPN 1): results of a double-blind, randomized, placebo-controlled, proof-of-concept study. Diabetes Care. (2024) 47:1325–32. 10.2337/dc24-0188 38895916 PMC11272977

[3] Borbjerg MK Wegeberg AM Nikontovic A Mørch CD Arendt-Nielsen L Ejskjaer N . Understanding the impact of diabetic peripheral neuropathy and neuropathic pain on quality of life and mental health in 6,960 people with diabetes. Diabetes Care. (2025) 48:588–95. 10.2337/dc24-2287 39932781

[4] Zhang Y Zhang H Wang K Liu X Li Z. Can spinal cord stimulation be considered as a frontier for chronic pain in diabetic foot? Pain Ther. (2025) 14:589–616. 10.1007/s40122-025-00710-0 39910016 PMC11914475

[5] Finnerup NB Attal N Haroutounian S McNicol E Baron R Dworkin RH . Pharmacotherapy for neuropathic pain in adults: a systematic review and meta-analysis. Lancet Neurol. (2015)14:162–73. 10.1016/S1474-4422(14)70251-0 25575710 PMC4493167

[6] Jang HN Oh TJ. Pharmacological and nonpharmacological treatments for painful diabetic peripheral neuropathy. Diabetes Metab J. (2023) 47:743–56. 10.4093/dmj.2023.0018 37670573 PMC10695723

[7] Rosenberger DC Blechschmidt V Timmerman H Wolff A Treede RD. Challenges of neuropathic pain: focus on diabetic neuropathy. J Neural Transm. (2020) 127:589–624. 10.1007/s00702-020-02145-7 32036431 PMC7148276

[8] Sloan G Selvarajah D Tesfaye S. Pathogenesis, diagnosis and clinical management of diabetic sensorimotor peripheral neuropathy. Nat Rev Endocrinol. (2021) 17:400–20. 10.1038/s41574-021-00496-z 34050323

[9] Ali R Schwalb JM. History and future of spinal cord stimulation. Neurosurgery. (2024) 94:20–8. 10.1227/neu.0000000000002654 37681953

[10] de Vos CC Meier K Zaalberg PB Nijhuis HJ Duyvendak W Vesper J . Spinal cord stimulation in patients with painful diabetic neuropathy: a multicentre randomized clinical trial. Pain. (2014)155:2426–31. 10.1016/j.pain.2014.08.031 25180016

[11] Taylor RS. Health-related quality of life and spinal cord stimulation in painful diabetic neuropathy. Diabetes Res Clin Pract. (2023) 206:110826. 10.1016/j.diabres.2023.110826 38245322

[12] Henson JV Varhabhatla NC Bebic Z Kaye AD Yong RJ Urman RD . Spinal cord stimulation for painful diabetic peripheral neuropathy: a systematic review. Pain Ther. (2021) 10:895–908. 10.1007/s40122-021-00282-9 34244979 PMC8586096

[13] Vallejo R Bradley K Kapural L. Spinal cord stimulation in chronic pain: mode of action. Spine. (2017) 14:S53–60. 10.1097/BRS.0000000000002179 28368982

[14] Heijmans L Joosten EA. Mechanisms and mode of action of spinal cord stimulation in chronic neuropathic pain. Postgrad Med. (2020) 132:17–21. 10.1080/00325481.2020.1769393 32403963

[15] Joosten EA Franken G. Spinal cord stimulation in chronic neuropathic pain: mechanisms of action, new locations, new paradigms. Pain. (2020) 161:S104–13. 10.1097/j.pain.0000000000001854 33090743 PMC7434213

[16] Wang ZB Liu YD Wang S Zhao P. High-frequency spinal cord stimulation produces long-lasting analgesic effects by restoring lysosomal function and autophagic flux in the spinal dorsal horn. Neural Regen Res. (2022) 17:370–7. 10.4103/1673-5374.317989 34269212 PMC8463971

[17] Bicket MC Dunn RY Ahmed SU. High-frequency spinal cord stimulation for chronic pain: pre-clinical overview and systematic review of controlled trials. Pain Med. (2016) 17:2326–36. 10.1093/pm/pnw156 28025366

[18] Chen P Jiang X Fu J Ou C Li Y Jia J . The potential mechanism of action of gut flora and bile acids through the TGR5/TRPV1 signaling pathway in diabetic peripheral neuropathic pain. Front Endocrinol. (2024) 15:1419160. 10.3389/fendo.2024.1419160 39619328 PMC11604420

[19] Tesfaye S Sloan G Petrie J White D Bradburn M Julious S . Comparison of amitriptyline supplemented with pregabalin, pregabalin supplemented with amitriptyline, and duloxetine supplemented with pregabalin for the treatment of diabetic peripheral neuropathic pain (OPTION-DM): a multicentre, double-blind, randomised crossover trial. Lancet. (2022) 400:680–90. 10.1016/S0140-6736(22)01472-6 36007534 PMC9418415

[20] Colloca L Ludman T Bouhassira D Baron R Dickenson AH Yarnitsky D . Neuropathic pain. Nat Rev Dis Primers. (2017) 3:17002. 10.1038/nrdp.2017.2 28205574 PMC5371025

[21] Yu J Wong S Lin Z Shan Z Fan C Xia Z . High-frequency spinal stimulation suppresses microglial Kaiso-P2X7 receptor axis-induced inflammation to alleviate neuropathic pain in rats. Ann Neurol. (2024) 95:966–83. 10.1002/ana.26898 38450773

[22] Provenzano DA Park N Edgar D Bovinet C Tate J. High-frequency (10 kHz) spinal cord stimulation (SCS) as a salvage therapy for failed traditional SCS: a narrative review of the available evidence. Pain Pract. (2023) 23:301–12. 10.1111/papr.13184 36409060

[23] Patel NP Wu C Lad SP Jameson J Kosek P Sayed D . Cost-effectiveness of 10-kHz spinal cord stimulation therapy compared with conventional medical management over the first 12 months of therapy for patients with nonsurgical back pain: randomized controlled trial. J Neurosurg Spine. (2023) 38:249–57. 10.3171/2022.9.SPINE22416 36272125

[24] Kapural L Sayed D Kim B Harstroem C Deering J. Retrospective assessment of salvage to 10 kHz spinal cord stimulation (SCS) in patients who failed traditional SCS therapy: RESCUE study. J Pain Res. (2020) 13:2861–7. 10.2147/JPR.S281749 33204147 PMC7667504

[25] Canós-Verdecho A Abejón D Robledo R Izquierdo R Bermejo A Gallach E . Randomized prospective study in patients with complex regional pain syndrome of the upper limb with high-frequency spinal cord stimulation (10-kHz) and low-frequency spinal cord stimulation. Neuromodulation. (2021) 24:448–58. 10.1111/ner.13358 33462918

[26] Petersen EA Stauss TG Scowcroft JA Jaasma MJ Edgar DR White JL . High-frequency 10-kHz spinal cord stimulation provides long-term (24-Month) improvements in diabetes-related pain and quality of life for patients with painful diabetic neuropathy. J Diabetes Sci Technol. (2024) 6:19322968241268547. 10.1177/19322968241268547 39369310 PMC11571607

[27] Tiago LMP Santos DFD Antunes DE Tiago LMP Goulart IMB. Assessment of neuropathic pain in leprosy patients with relapse or treatment failure by infrared thermography: a cross-sectional study. PLoS Negl Trop Dis. (2021) 15:e0009794. 10.1371/journal.pntd.0009794 34555035 PMC8491942

